# Identification of Biomarkers Associated With Pathological Stage and Prognosis of Clear Cell Renal Cell Carcinoma by Co-expression Network Analysis

**DOI:** 10.3389/fphys.2018.00399

**Published:** 2018-04-18

**Authors:** Liang Chen, Lushun Yuan, Kaiyu Qian, Guofeng Qian, Yuan Zhu, Chin-Lee Wu, Han C. Dan, Yu Xiao, Xinghuan Wang

**Affiliations:** ^1^Department of Urology, Zhongnan Hospital of Wuhan University, Wuhan, China; ^2^Department of Biological Repositories, Zhongnan Hospital of Wuhan University, Wuhan, China; ^3^Laboratory of Precision Medicine, Zhongnan Hospital of Wuhan University, Wuhan, China; ^4^Department of Endocrinology, The First Affiliated Hospital of Zhejiang University, Hangzhou, China; ^5^Department of Urology, Massachusetts General Hospital, Harvard Medical School, Boston, MA, United States; ^6^Greenebaum Cancer Center, School of Medicine, University of Maryland, Baltimore, MD, United States

**Keywords:** clear cell renal cell carcinoma (ccRCC), differentially expressed genes (DEGs), weighted gene co-expression network analysis (WGCNA), survival prognosis, pathological stage

## Abstract

Clear cell renal cell carcinoma (ccRCC) is the most common subtype among renal cancer whose prognostic is affected by the tumor progression associated with complex gene interactions. However, there is currently no available molecular markers associated with ccRCC progression and used or clinical application. In our study, microarray data of 101 ccRCC samples and 95 normal kidney samples were analyzed and 2,425 differentially expressed genes (DEGs) were screened. Weighted gene co-expression network analysis (WGCNA) was then conducted and 11 co-expressed gene modules were identified. Module preservation analysis revealed that two modules (red and black) were found to be most stable. In addition, Pearson's correlation analysis identified the module most relevant to pathological stage(patho-module) (*r* = 0.44, *p* = 3e-07). Functional enrichment analysis showed that biological processes of the patho-module focused on cell cycle and cell division related biological process and pathway. In addition, 29 network hub genes highly related to ccRCC progression were identified from the stage module. These 29 hub genes were subsequently validated using 2 other independent datasets including GSE53757 (*n* = 72) and TCGA (*n* = 530), and the results indicated that all hub genes were significantly positive correlated with the 4 stages of ccRCC progression. Kaplan-Meier survival curve showed that patients with higher expression of each hub gene had significantly lower overall survival rate and disease-free survival rate, indicating that all hub genes could act as prognosis and recurrence/progression biomarkers of ccRCC. In summary, we identified 29 molecular markers correlated with different pathological stages of ccRCC. They may have important clinical implications for improving risk stratification, therapeutic decision and prognosis prediction in ccRCC patients.

## Introduction

Renal carcinoma is a common malignancy of the urinary system and accounts for 2–3% of adult malignancies (Siegel et al., 2017). Renal cell carcinoma (RCC) accounts for about 90% of renal cancers, the vast majority (70–85%) of which are clear cell subtypes (Cairns, 2010). RCC can be divided into 4 pathological stages according to tumor size, the extend of invasion and metastasis (Motzer et al., 2015). Surgery treatment is effective for localized RCC, however, once the RCC becomes metastatic the survival rate of patients will drop sharply. In addition, about 30% of patients with RCC had metastasis when diagnosed. The standard treatment for localized RCC is surgery including radical or partial nephrectomy due to its insensitive to radiotherapy and chemotherapy (Motzer et al., 1996). For metastatic RCC, immunotherapy with interleukin-2 (IL-2) and interferon (IFN) was once the standard treatment, but it had limited curative effects and strong side effects (Negrier et al., 1998). Targeted therapies including sorafenib (Hutson et al., 2010) and sunitinib (Motzer et al., 2006) were approved for metastatic RCC in 2005 and 2006 respectively, with better effects and fewer side effects compared with immunotherapy. However, targeted therapies were still limited and prone to drug resistance (Coppin et al., 2011; Ljungberg et al., 2015). Therefore, more effective diagnosis biomarkers and therapeutic targets are in urgent need.

Gene expression profiles such as microarray and RNA-sequencing have been widely used to identify biomarkers associated with clear cell renal cell carcinoma (ccRCC) progression (Dahinden et al., 2010; Gerlinger et al., 2014). However, most of the published studies focused on the screening of differently expressed genes (DEGs), ignoring the high correlations between genes, although genes with similar expression patterns might be functionally related (Tavazoie et al., 1999). Weighted gene co-expression network analysis (WGCNA) was applied to explore the correlations between gene clusters and clinical features (Langfelder and Horvath, 2008). Recently, many studies related to WGCNA regarding biological information and systems biology have been published in well-known journals (Kunowska et al., 2015; Luo et al., 2015). The WGCNA algorithm had been applied to screen for biological processes and treatment targets of cancer as well as specific biomarkers related to complex disease, such as familial combination of hyperlipidemia (Plaisier et al., 2009), Alzheimer's disease (Miller et al., 2010) and osteoporosis (Farber, 2010). Similarly, WGCNA was also used to identify key genes significantly correlated with clinical indicators of tumor progression including tumor stages, grades and metastasis for different tumor types (Chen P. et al., 2017; He et al., 2017).

Thus, our study aims to identify network-centric genes associated with ccRCC progression by constructing a co-expression network using DEGs through weighted gene co-expression network WGCNA (Clarke et al., 2013; Chou et al., 2014). To our knowledge, it is the first attempt to use WGCNA to identify a series of hub genes as biomarkers significantly associated with pathological stages and prognosis of ccRCC and to distinguish localized and non-localized ccRCC.

## Materials and methods

### Data collection

Raw gene expression profile and clinical data were obtained from Gene Expression Omnibus (GEO) database (http://www.ncbi.nlm.nih.gov/geo/). Datasets GSE53757 Von Roemeling et al., 2014 and GSE36895 (Peña-Llopis et al., 2012) performed on the same platform Affymetrix Human Genome U133 Plus 2.0 Array (HG U133 Plus 2.0) were combined and analyzed to screen DEGs. Dataset GSE73731 (Wei et al., 2017) was also performed on the same platform included 265 ccRCC samples, of which 125 samples with complete clinical data were used to identify hub genes with WGCNA. Two other independent datasets GSE40355 based on the platform of Whole Human Genome Microarray 4 × 44 K v2 (Agilent-026652) and GSE36895 were used to conduct module preservation analysis. In addition, RNA sequencing data of 530 ccRCC samples was downloaded from The Cancer Genome Atlas (TCGA) database (https://genome-cancer.ucsc.edu/) for further validation. Gene expression data was based on Illumina Hiseq's RNA sequencing technology. The detailed information of the datasets used in this study was summarized in Supplementary Table S1.

### Data preprocessing and differentially expressed genes (DEGs) screening

Raw expression data was calculated following the pre-processing procedures: RMA background correction, log2 transformation, quantile normalization and median polish algorithm summarization using the “affy” (Gautier et al., 2004) package of R software (version 3.3.1). Besides, “sva” (Leek and Storey, 2007) R package was used to remove batch effects between dataset GSE53757 and GSE36895. Probes were annotated by the Affymetrix annotation files. R package “limma” (Ritchie et al., 2015) was applied to select the DEGs between 101 ccRCC samples and 95 normal kidney samples. The cut-off criteria for screening DEGs was the false discovery rate (FDR) <0.01 and |log2(fold change)| ≥ 1.

### Weighted gene co-expression network construction

The “WGCNA” (Langfelder and Horvath, 2008) package in R was applied to performed co-expression network using the expression values of 2,425 DEGs from 125 tumor samples and complete clinical data (GSE73731). First, one outlier sample was excluded from subsequent analysis (Supplementary Figure S2). The detailed procedure for WGCNA construction could be found in our previous study (Chen L. et al., 2017). Briefly, we constructed the weighted adjacency matrix using a power function based on a soft-thresholding parameter β. After that, the adjacency was transformed into topological overlap matrix (TOM), and average linkage hierarchical clustering was performed according to the TOM-based dissimilarity measure. In this study, we chose a minimum size (gene group) of 30 for the genes dendrogram and a cut-line (0.25) for module dendrogram and merged some modules.

### Module preservation analysis

To access the stability of the each module identified above, we conducted module preservation analysis using the modulePreservation (Langfelder et al., 2011) method (nPermutations = 200) in the “WGCNA” package. The two datasets GSE36895 and GSE40355 used for preservation analysis contained gene expression profiles of 29 and 16 ccRCC samples, respectively.

### Identifying clinically significant modules and module functional annotation

WGCNA identifies gene modules based on their expression similarities in samples and calculates the correlation between the external clinical information and gene modules to identify clinically significant gene modules. The gene modules most correlated with clinical features were selected as modules of interest, combined with the correlative clinical feature. To further clarify the mechanism underlying the impact of module genes on correlative clinical feature, genes in interest module were uploaded to the Database for Annotation, Visualization, and Integrated Discovery (DAVID) (Dennis et al., 2003) for GO functional annotation and KEGG pathway enrichment analysis. FDR < 0.05 was used as cut-off criteria.

### Hub genes identification and validation

Hub genes were a series of genes with the highest degree of connectivity in a gene module and determined the characteristics of a module. Hub genes were defined by module connectivity, measured by absolute value of the Pearson's correlation (cor.geneModuleMembership > 0.8) and clinical trait relationship, and measured by absolute value of the Pearson's correlation (cor.geneTraitSignificance > 0.2). In this study, we identified hub genes in the module which significantly correlated with certain clinical feature. In addition, we used boxplot to show the relationship between the hub genes and the corresponding clinical features, and the statistical significance between them was analyzed by one-way ANOVA.

Another independent dataset GSE53757 was analyzed to validate the hub genes. In addition, 530 ccRCC samples from TCGA database were analyzed to compare expression of hub genes between different pathological stages of ccRCC.

### Survival analysis

In order to evaluate the impact of all hub genes on ccRCC patient's prognosis, overall and disease-free survival were analyzed. For overall survival analysis, 530 patients were divided into 2 groups according to median expression of each hub gene (high vs. low). Similarly, for disease free survival, 433 patients with complete recurrence/progression survival time were also analyzed. Then we adopted “survival” of R software for log-rank test and Kaplan-Meier survival analysis (Goel et al., 2010).

## Results

### Identification of differentially expressed genes in ccRCC tissue samples

A workflow of this study was shown in Supplementary Figure S4. The expression matrices for 196 samples containing 101 ccRCC samples and 95 normal kidney samples in datasets GSE53757 and GSE36895 were obtained after data preprocessing. A total of 2,425 DEGs including 1,259 up-regulated and 1166 down-regulated were screened between ccRCC and normal kidney under the threshold of FDR < 0.01 and |log2FC| ≥ 1. These 2,425 DEGs were then selected for subsequent analysis. The heatmap for DEGs were shown in Supplementary Figure S1.

### Weighted co-expression network construction and module preservation analysis

**Figure 1:**
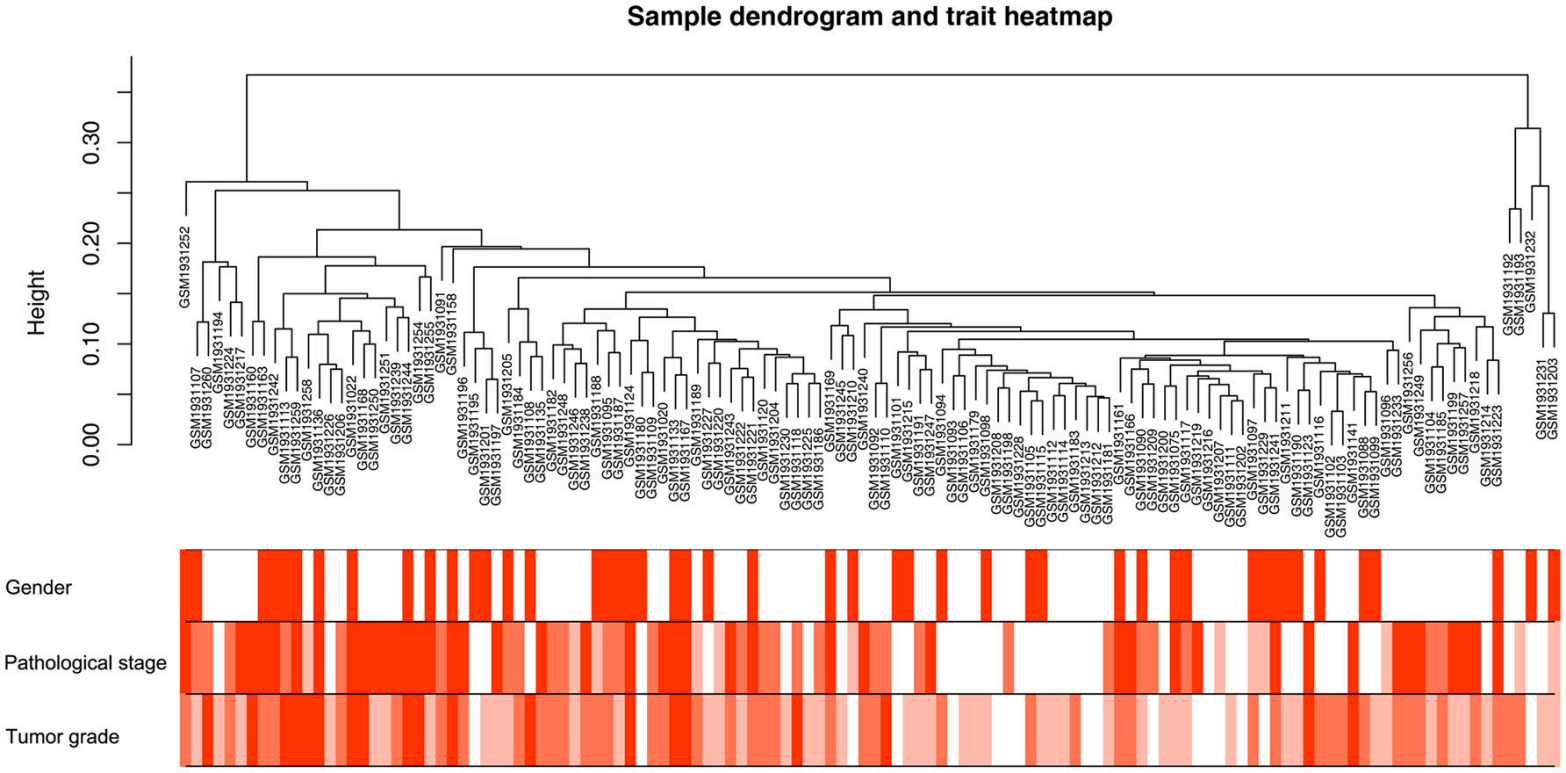
Clustering dendrogram of 124 tumor samples and the clinical traits. The clustering was based on the expression data of DEGs between tumor samples and non-tumor samples in ccRCC. The color intensity was proportional to older age as well as higher pathological stage and tumor grade.

**Figure 2:**
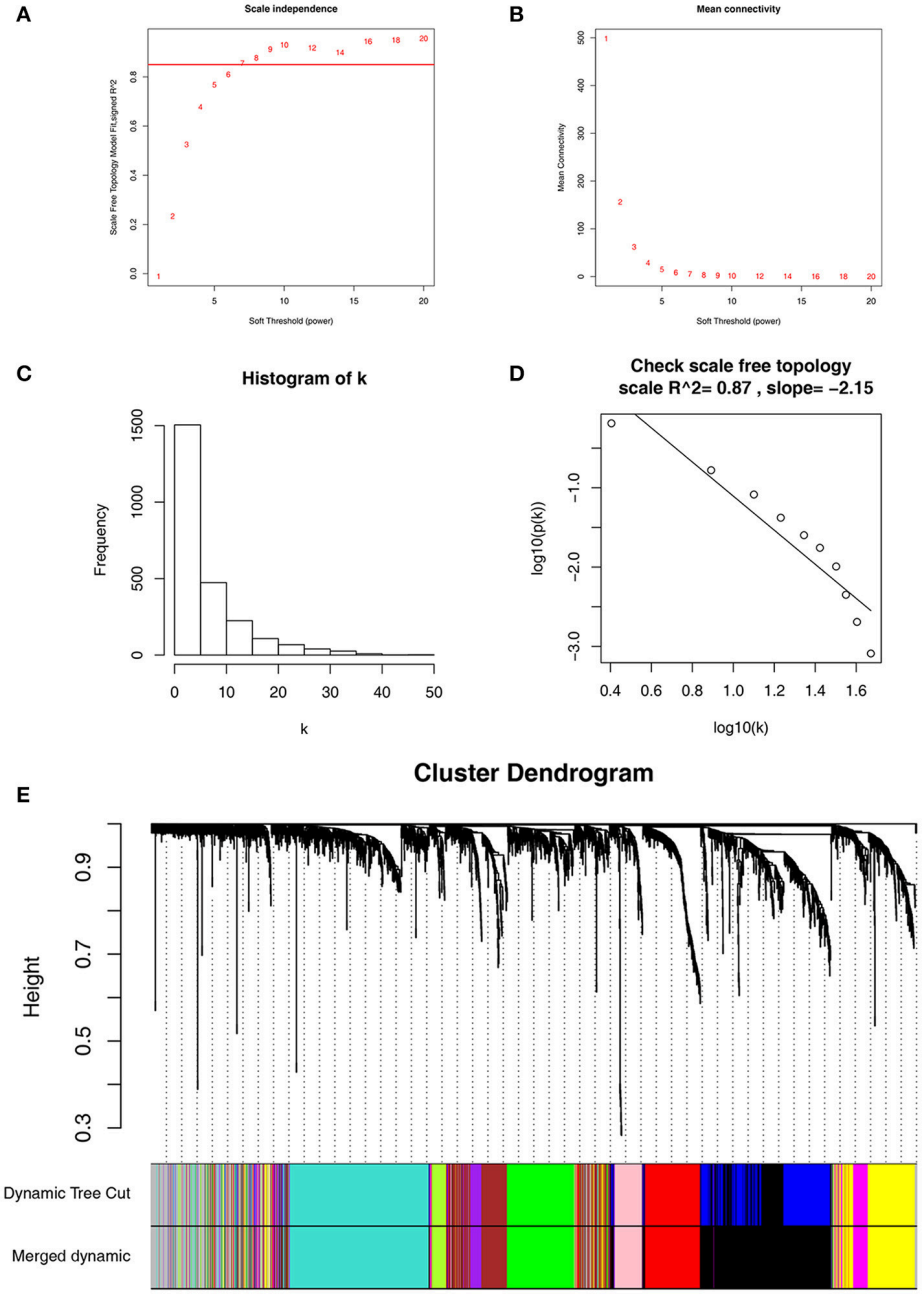
Determination of soft-thresholding power in WGCNA. **(A)** Analysis of the scale-free fit index for various soft-thresholding powers (β). **(B)** Analysis of the mean connectivity for various soft-thresholding powers. **(C)** Histogram of connectivity distribution when β = 7. **(D)** Checking the scale free topology when β = 7. **(E)** Dendrogram of all differentially expressed genes clustered based on a dissimilarity measure (1-TOM).

Co-expression network was constructed using independent dataset GSE73731 including 124 ccRCC samples associated with complete clinical data (Figure 1). The expression values of 2,425 DEGs were included for co-expression network constructing by adopting “WGCNA” package. In current study, to ensure a scale-free network, we selected β = 7 (scale free *R*^2^ = 0.87) as the soft-thresholding power (Figures 2A–D), and identified a total of 11 modules (Figure 2E).

**Figure 3:**
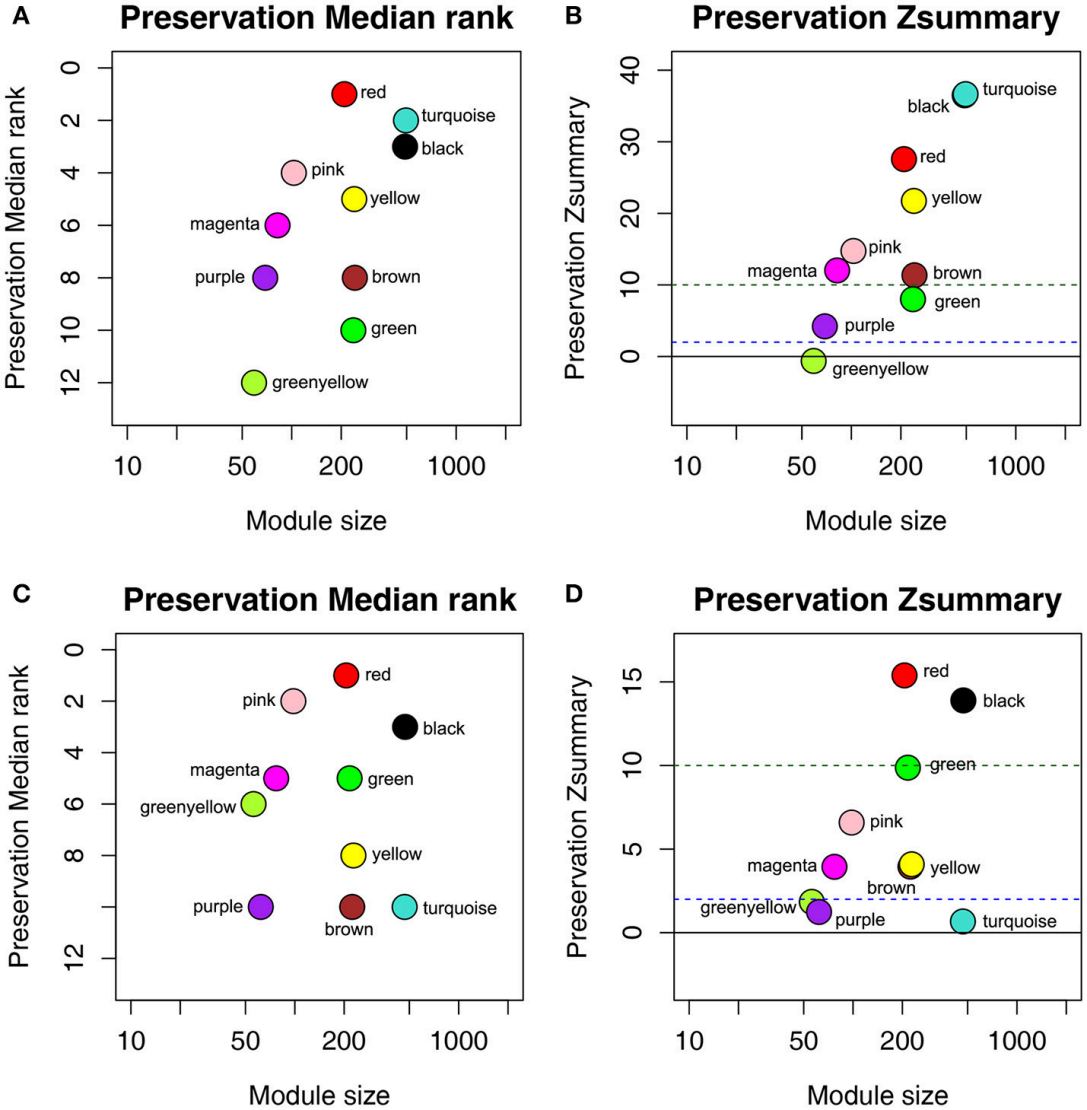
The medianRank and Zsummary statistics of the module preservation of the DEG modules using two independent dataset GSE36895 **(A,B)** and GSE40355 **(C,D)**. The medianRank of the modules close to zero indicates a high degree of module preservation **(A,C)**. The dashed blue and green lines indicate the thresholds Z = 2 and Z = 10, respectively **(B,D)**. These horizontal lines indicate the Zsummary thresholds for strong evidence of conservation (above 10) and for low to moderate evidence of conservation (above 2).

To determine whether the identified network can also be found in another independent network, we performed module preservation analysis by comparing the GSE73731 dataset with 2 other test datasets GSE36895 and GSE40355. As shown in Figure 3, red and black modules were found to be most stable due to their Zsummary statistics (Figures 3B,D) above 10 and Median rank statistics (Figures 3A,C) close to minimum both in the two test datasets.

### Identification of key modules and functional annotation

**Figure 4:**
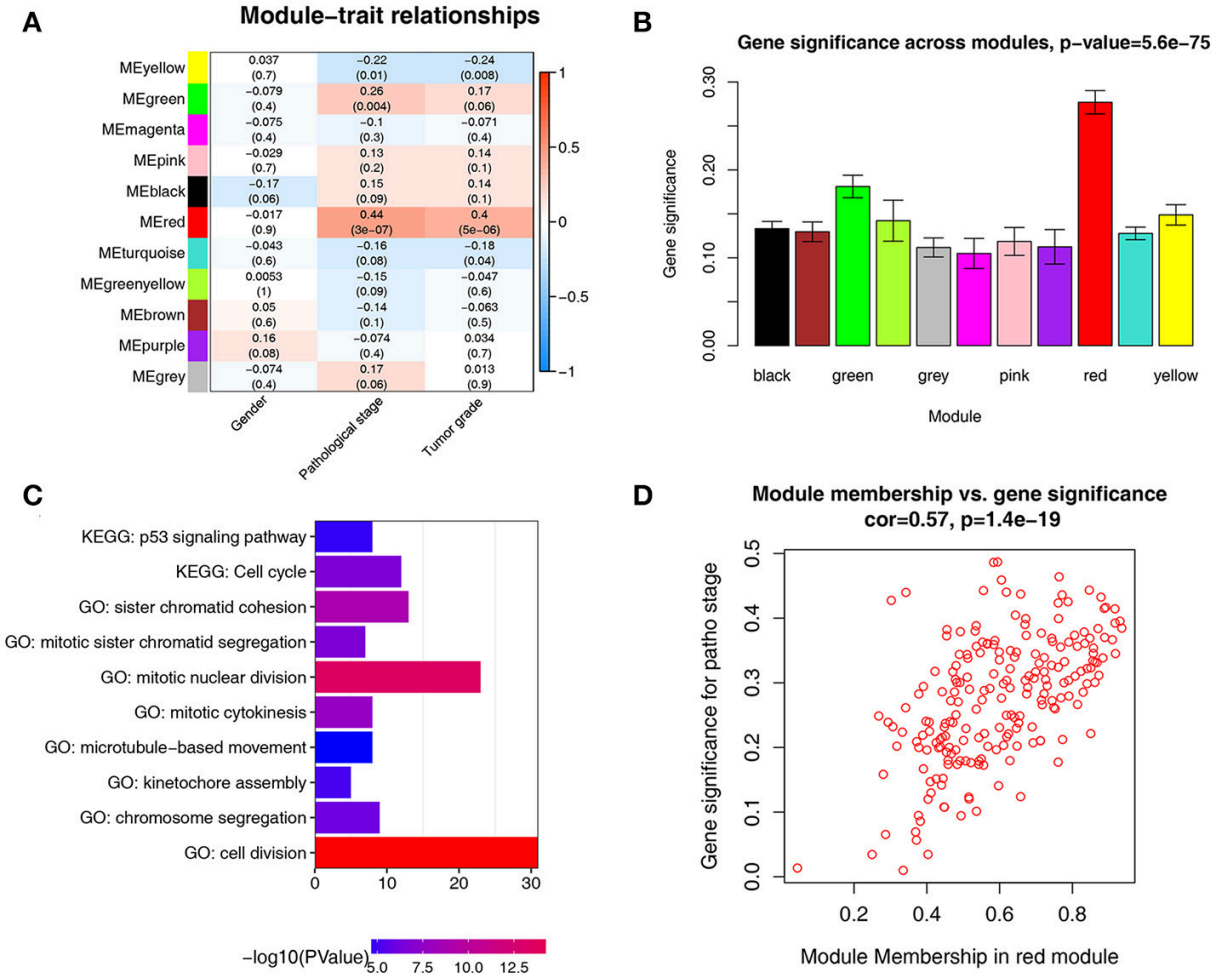
Identification of modules associated with the clinical traits of ccRCC. **(A)** Heatmap of the correlation between module eigengenes and clinical traits of ccRCC. **(B)** Distribution of average gene significance and errors in the modules associated with pathological stage of ccRCC. **(C)** GO functional and KEGG pathway enrichment analyses for genes in the red module. The –log10 (*P*-value) of each term is colored according to the legend. **(D)** Scatter plot for correlation between gene module membership in the red module(patho-module) and gene significance.

There were great biological implications to identify modules most significantly related to clinical features. We found that red module showed the highest correlation with pathological stage (*r* = 0.44, *p* = 3e-7, Figure 4A). We defined the module most relevant to pathological stage(red) as patho-module.

Besides, in relation with pathological stage, the patho-module also exhibited the highest gene significance (Figure 4B). Therefore, we selected the patho-module as module of interest and analyzed subsequently. Interestingly, the patho-module also showed high correlation with tumor grade. The genes in each module were listed in Supplementary Table S2.

To figure out the functional involvement of the patho-module, 209 genes in patho-module were uploaded into DAVID database for Gene Ontology (GO) analysis and KEGG pathway enrichment analysis. Under the threshold of FDR < 0.05, biological processes of patho-module were suggested to focus on cell division (*p* = 1.24e-18), mitotic nuclear division (*p* = 3.61e-14), sister chromatid cohesion (*p* = 1.26E-09), mitotic cytokinesis (*p* = 2.07e-08), mitotic sister chromatid segregation (*p* = 2.30e-07), chromosome segregation (*p* = 7.22e-07), kinetochore assembly (*p* = 6.31e-06) and microtubule-based movement (*p* = 2.86e-05, Figure 4C). While KEGG pathway enrichment analysis suggested that 209 genes in patho-module was significantly enriched in two pathways including cell cycle (2.15e-07) and p53 signaling pathway (*p* = 1.36e-05, Figure 4C).

### Identification of hub genes

Under the threshold of module connectivity (cor.geneModuleMembership) more than 0.8 and clinical trait relationship (cor.geneTraitSignificance) more than 0.2, 29 genes with the high connectivity in patho-module were selected as hub genes (Figure 4D). Hub genes were significantly correlated with pathological stage were listed in Table 1.

**Table 1:** Hub genes in the modules related with pathological stages.

**Gene**	**Entrez ID**	**Probe ID**	**Co-expression analysis**		**DEG analysis**	
			**cor.geneModuleMembership**	**cor.gene TraitSignificance**	**Fold change**	**FDR**
ANLN	54443	222608_s_at	0.918	0.345	4.33	0
ASPM	259266	219918_s_at	0.870	0.383	4.95	0
ATAD2	29028	222740_at	0.813	0.356	2.57	0
BUB1B	701	203755_at	0.916	0.414	2.61	0
CCNA2	890	213226_at	0.872	0.311	2.57	0
CCNB1	891	214710_s_at	0.887	0.415	2.20	0
CDK1	983	203213_at	0.850	0.221	2.28	0
CDKN3	1033	1555758_a_at	0.813	0.266	2.36	0
CENPF	1063	207828_s_at	0.861	0.332	2.49	0
CENPK	64105	222848_at	0.840	0.322	4.19	0
CENPW	387103	226936_at	0.805	0.373	2.01	0
CEP55	55165	218542_at	0.935	0.384	2.88	0
DLGAP5	9787	203764_at	0.840	0.298	2.63	0
FOXM1	2305	202580_x_at	0.832	0.378	2.03	0
KIF11	3832	204444_at	0.855	0.395	2.61	0
KIF14	9928	236641_at	0.930	0.333	2.11	0
KIF20A	10112	218755_at	0.847	0.368	3.08	0
KIF4A	24137	218355_at	0.883	0.338	2.42	0
MELK	9833	204825_at	0.850	0.323	2.23	0
NCAPG	54892	218662_s_at	0.823	0.317	2.12	0
NEK2	4751	204641_at	0.857	0.345	2.41	0
NUF2	83540	223381_at	0.891	0.416	2.30	0
PRC1	9055	218009_s_at	0.909	0.366	4.01	0
PTTG1	9232	203554_x_at	0.829	0.285	2.76	0
RRM2	6241	201890_at	0.888	0.370	5.29	0
TOP2A	7153	201291_s_at	0.917	0.392	5.59	0
TPX2	22974	210052_s_at	0.833	0.360	2.91	0
TTK	7272	204822_at	0.846	0.443	2.55	0
UHRF1	29128	225655_at	0.867	0.331	3.65	0

**Figure 5:**
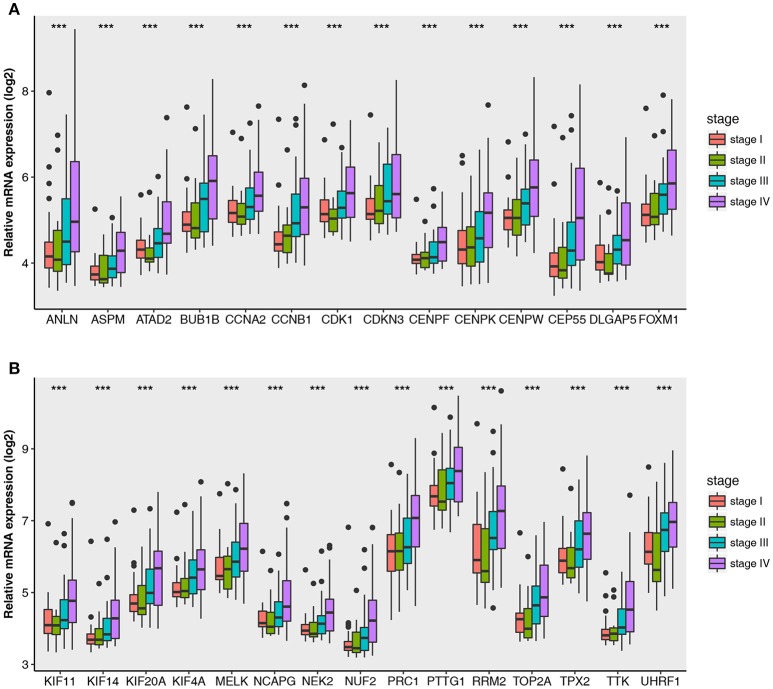
Boxplots of hub genes across different pathological stages in the GSE73731. The boxplots show the medians and dispersions of the samples of different pathological stages for each hub gene. **(A)** Boxplots of hub genes from ANLN to FOXM1 (sort in alphabetical order) in different pathological stages. **(B)** Boxplots of hub genes from KIF11 to UHRF1 in different pathological stages. *P*-values are the results of one-way ANOVA for different pathological stages. ***p < 0.001.

The relationship between all hub gene and pathological stages in the training dataset GSE73731 was shown in Figure 5. Expression values of all hub genes in different pathological stages were compared and statistical differences were calculated with one-way ANOVA, suggesting significant difference (*p* < 0.01) of each hub gene across different pathological stages. Interestingly, we also found significant difference (*p* < 0.01) of each hub gene across different tumor grades (Supplementary Figure S3).

### Validation of hub genes

**Figure 6:**
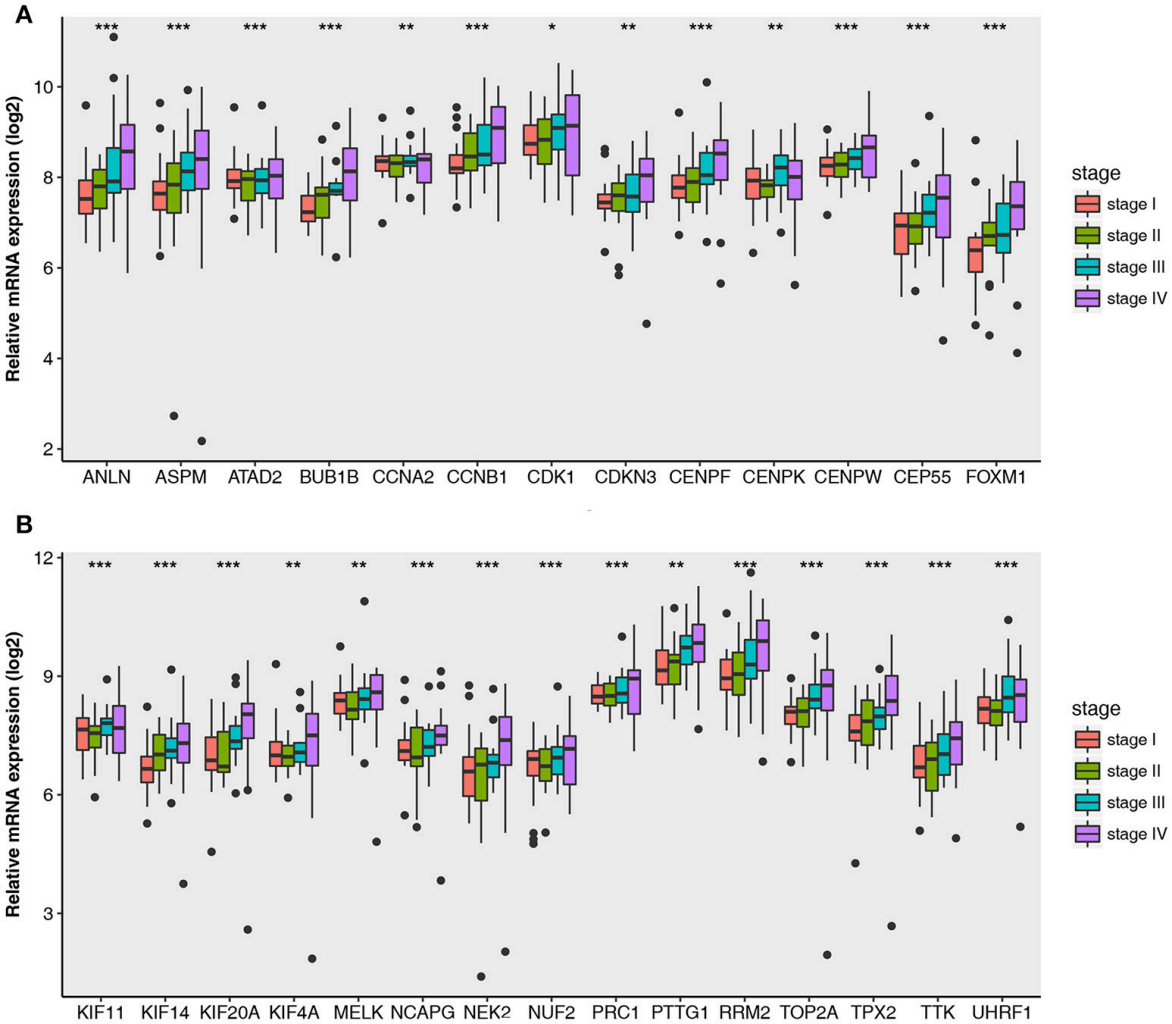
Boxplots of hub genes across different pathological stages in the validation dataset GSE53757. The boxplots show the medians and dispersions of the samples of different pathological stages for each hub gene. **(A)** Boxplots of hub genes from ANLN to FOXM1 (sort in alphabetical order) in different pathological stages. **(B)** Boxplots of hub genes from KIF11 to UHRF1 in different pathological stages. *P*-values are the results of one-way ANOVA for different pathological stages. *p < 0.05, **p < 0.01, ***p < 0.001.

**Figure 7:**
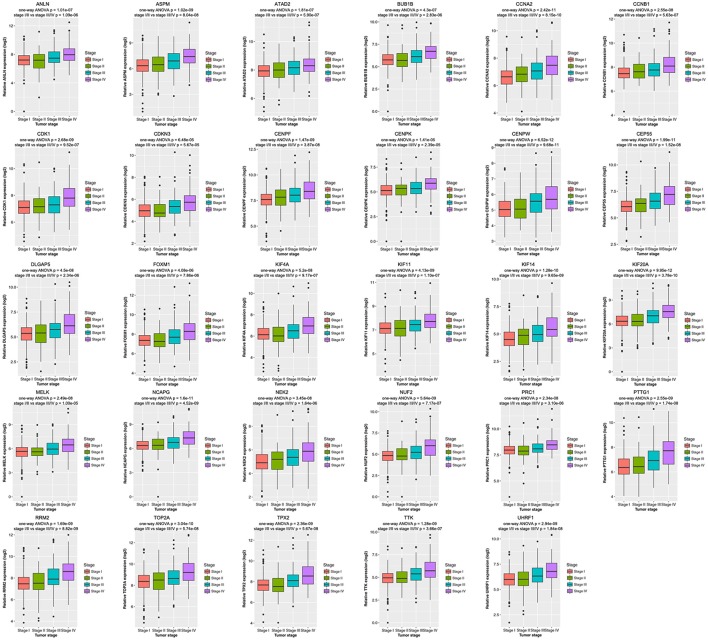
Boxplots of hub genes across different pathological stages in the TCGA dataset. The boxplots show the medians and dispersions of the samples of different pathological stages for each hub gene. *P*-values are the results of independent sample *t*-test between pathological stage I/II and III/IV and one-way ANOVA for different pathological stages.

Then all hub genes were selected for validation using 2 other independent datasets including GSE53757 and TCGA dataset. In the test set GSE53757, significant difference was detected for each hub gene expression across 4 pathological stages using one-way ANOVA (Figure 6). Moreover, one-way ANOVA and independent sample *t*-tests based on RNA sequencing data showed that all hub genes were also effective to distinguish local ccRCC (pathological stage I or II) and non-localized ccRCC (pathological stage III or IV, Figure 7).

### Survival analysis

**Figure 8:**
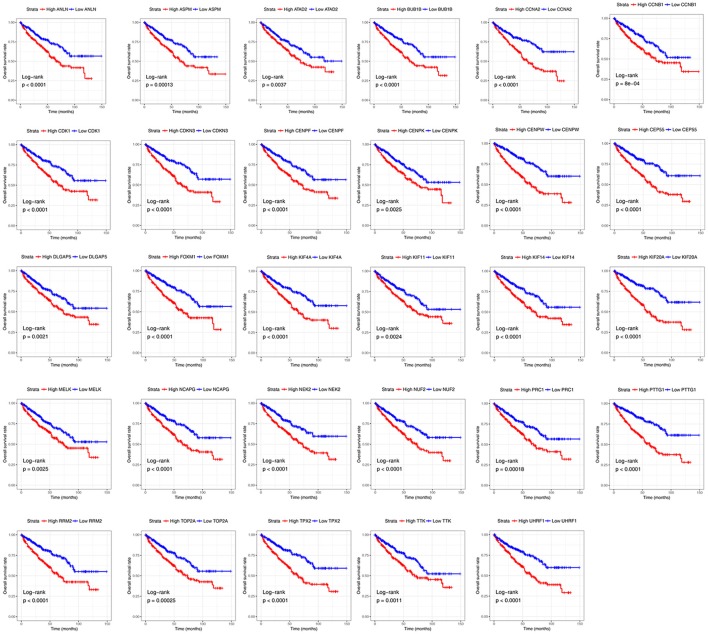
Overall survival analyses on hub genes in the TCGA data set. Survival curves for patients in different groups. Red lines represent high expression of hub genes, while blue lines represent low expression of hub genes.

**Figure 9:**
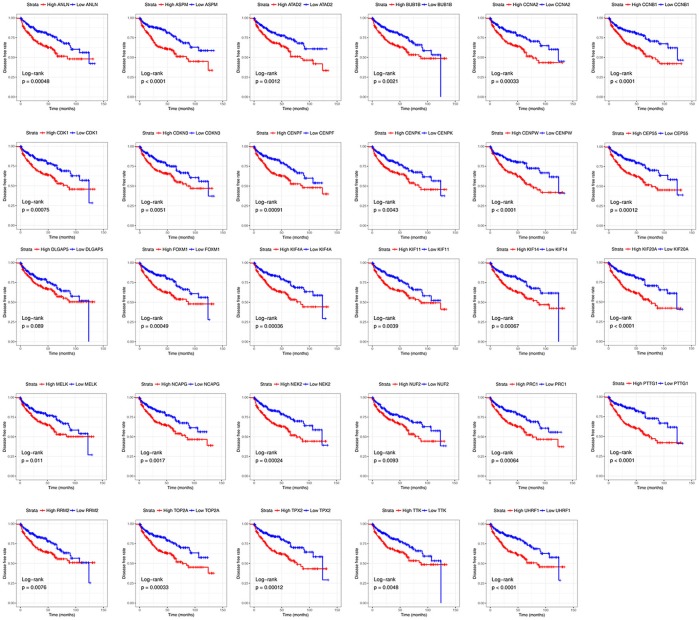
Diseases-free survival analyses on hub genes in the TCGA data set. Survival curves for patients in different groups. Red lines represent high expression of hub genes, while blue lines represent low expression of hub genes.

Base on TCGA RNA-sequencing data and clinical information, patients were divided into 2 groups according to median expression of each hub gene, and Kaplan-Meier survival curve was then plotted. Patients with higher each hub gene showed significantly shorter overall survival rate and disease-free survival rate, indicating that all hub genes could act as prognosis and recurrence biomarkers of ccRCC (Figures 8, 9).

## Discussion

ccRCC is the most common subtype among renal cancer whose prognostic is affected by the tumor progression associated with complex gene interactions. Exploring molecular markers of ccRCC is important for the diagnosis and treatment of patients with ccRCC. However, there is currently no available molecular markers associated with the pathological stage of ccRCC for clinical application. Here, clinical information and mRNA expression profiling of patients with ccRCC from microarray data were analyzed by using a systematic biological method WGCNA. We identified 29 molecular markers correlated with different pathological stages of ccRCC. They may have important clinical implications for improving risk stratification, therapeutic decision-making and prognosis prediction in patients with ccRCC.

WGCNA, an algorithm to mine gene module information from expression profiling microarray, was widely applied in the RNA-sequencing data (Langfelder and Horvath, 2008). In this method, module was defined as a group in which genes had similar expression variation trend in a physiological process or in different tissue samples. In another word, genes with similar expression variation trend across different samples could be defined as a gene module. The WGCNA clustering criteria had a great biological significance which was completely different from the clustering method based on the geometric distance between data. After the gene module was identified, the stability of module was calculated. Then the correlation between stable modules and clinical features such as age, gender, stage of disease, and tumor grade were calculated. By this method, the most clinically related gene modules which could be used to explore the primary cause of disease development. Scale-free network was characterized by the existence of a few nodes that have significantly higher connectivity than the general nodes. The “few” node genes were defined as hub genes. So, the studies for the correlation between interest module and certain clinical feature could be simplified as correlation between hub genes of interest module and clinical feature, and thus providing an important molecular basis for exploring the mechanism of disease development.

The aim of the study was to identify biomarkers associated with progression of ccRCC using WGCNA to mine expression profiling combined with clinical data of large numbers of patients with ccRCC. In the study of cancer, candidate molecular biomarkers must be well distinguished between cancerous and normal tissues. Based on DEGs screened from ccRCC, the weighted co-expression network was constructed and 11 co-expression modules were identified through dynamic tree cutting method. It was found that among the 11 modules the red one showed the highest positive correlation with tumor pathological stage by correlation analysis. In addition, the preservation statistics suggested that the stability of the patho-module is good. Therefore, the patho-module was considered as a clinically significant gene cluster. Hub genes with the highest connectivity in a gene module largely decided the characteristics of the module.

Functional annotation of patho-module was suggested to focus on cell division, cell cycle, mitotic nuclear division, sister chromatid cohesion, mitotic cytokinesis, etc. Cell cycle and cell division are the basic process of cell proliferation, the abnormal mediation of which will lead to the tumor progression. In current study, 29 hub genes of patho-module significantly correlated with pathological stage were identified and validated, which could distinguish localized (pathological stage I/II) and non-localized ccRCC (pathological stage III/IV). Interestingly, expression value of each hub gene across different tumor grades could also be found significant difference, suggesting that the 29 hub genes were positively correlated with tumor grade of ccRCC as well. These findings may contribute to the improvement of therapeutic decision-making, risk stratification, and prognosis prediction for ccRCC patients.

Previous studies using TCGA ccRCC data accurately predicted molecular markers associated with histological grading of ccRCC. Wan et. al identified 8 genes that could be used to distinguish different grades of ccRCC by comparing different histological grades of ccRCC (grade I/II vs. grade III/IV) (Wan et al., 2017). However, this analysis method did not utilize a global level system biological analysis method, which might cause large false-positive results. Other studies using the collected tissue samples for immunohistochemical analysis showed that EphA1 (Wang L. et al., 2015), EphA2 (Wang X. et al., 2015), and VEGFR-1 (Lkhagvadorj et al., 2014) were associated with different pathological stages of ccRCC by directly comparing gene expression differences. However, the results lacked large sample support.

WGCNA is a method that can be applied to explore potential biological mechanisms and to identify genes associated with patient's prognosis. The practical utility of this approach can predict new prognostic markers. Our research may contribute to the personalized treatment of patients with ccRCC. Nonetheless, prior to the clinical use of these molecular markers, multiple center randomized controlled clinical trials and *in vitro/vivo* experiments should be conducted to assess the potential application of molecular features, to predict survival and to functionally characterize hub genes in clinical applications.

A larger number of clinical samples were required to validate our findings and elucidate the underlying mechanisms of how these hub genes impacted pathological stage of ccRCC, which were our subsequent research work.

In summary, we established a gene co-expression network to identify and validate network hub genes associated with the progression of ccRCC, based on systems biology-based WGCNA. Our work might have important clinical implications for improving risk stratification, therapeutic decision-making and prognosis prediction in patients with ccRCC.

## Author contributions

LC, LY, YX, and XW conceived and designed the study; LC, LY, KQ, and YX performed the analysis procedures; LC, GQ, YZ, HD, and YX analyzed the results; LC, C-LW, YX, and XW contributed analysis tools; LC, LY, and YX contributed to the writing of the manuscript. All authors reviewed the manuscript.

### Conflict of interest statement

The authors declare that the research was conducted in the absence of any commercial or financial relationships that could be construed as a potential conflict of interest.
