# Supplementary material for: Identification of Biomarkers Associated With Pathological Stage and Prognosis of Clear Cell Renal Cell Carcinoma by Co-expression Network Analysis
Source: Front Physiol. 2018 Apr 18;9:399. doi: 10.3389/fphys.2018.00399 (PMC5915556; doi:10.3389/fphys.2018.00399)
Supplement: Supplementary file 1 [file Image1.PDF]

Supplementary Figure S1

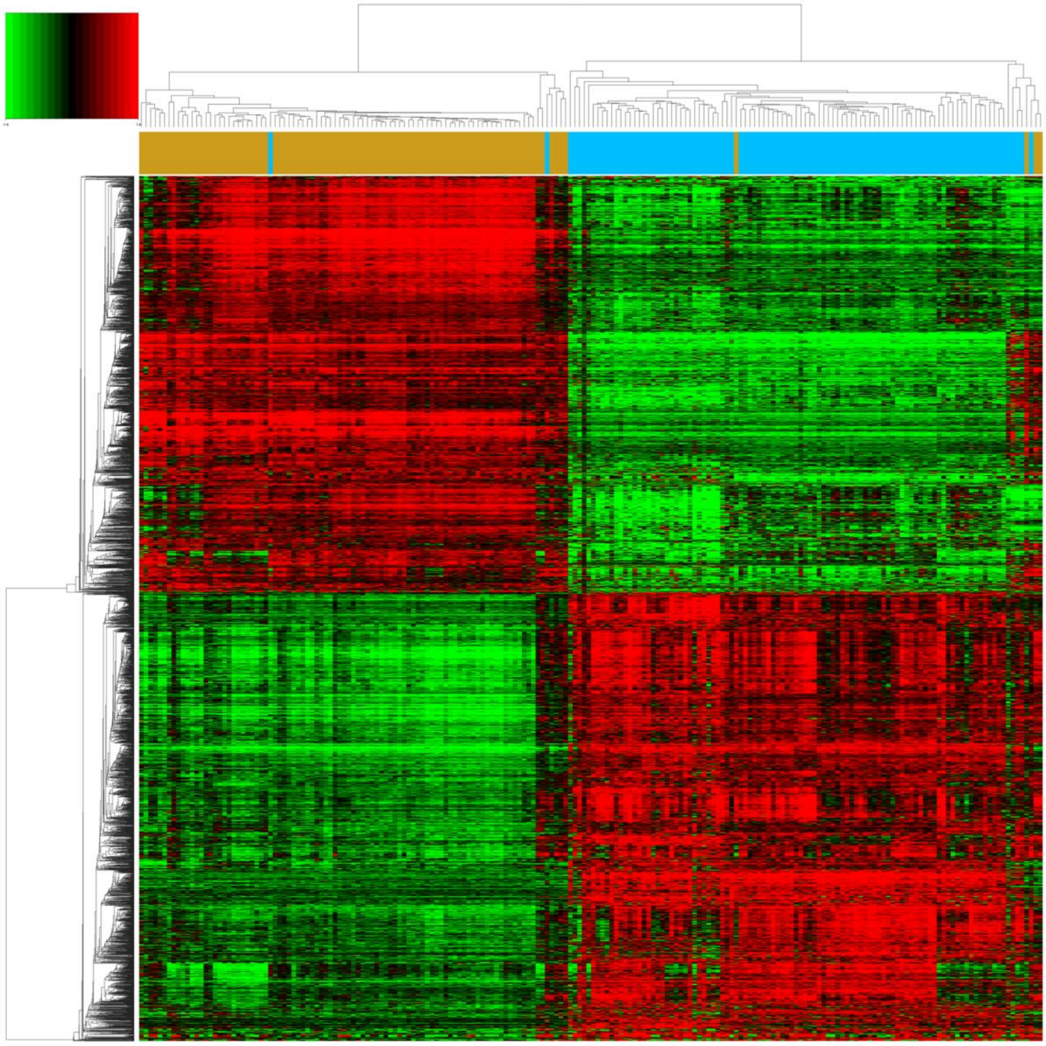

Supplementary Figure S1. Heatmap of differentially expressed genes identified from 101 ccRCC samples and 95 normal kidney samples.
