# Supplementary material for: Identification of Biomarkers Associated With Pathological Stage and Prognosis of Clear Cell Renal Cell Carcinoma by Co-expression Network Analysis
Source: Front Physiol. 2018 Apr 18;9:399. doi: 10.3389/fphys.2018.00399 (PMC5915556; doi:10.3389/fphys.2018.00399)
Supplement: Supplementary file 2 [file Image2.PDF]

Supplementary Figure S2

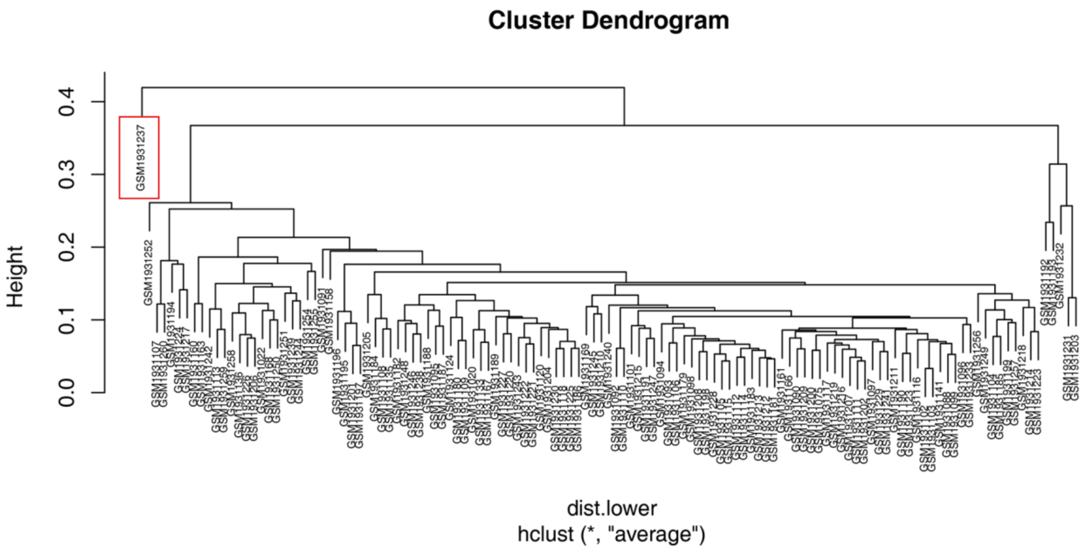

Supplementary Figure S2. Clustering analysis of 125 tumor samples with complete clinical data (GSE73731). One outlier sample was removed in order to ensure the results of network construction reliable.
