# Supplementary material for: Identification of Biomarkers Associated With Pathological Stage and Prognosis of Clear Cell Renal Cell Carcinoma by Co-expression Network Analysis
Source: Front Physiol. 2018 Apr 18;9:399. doi: 10.3389/fphys.2018.00399 (PMC5915556; doi:10.3389/fphys.2018.00399)
Supplement: Supplementary file 3 [file Image3.PDF]

## **Supplementary Figure S3**

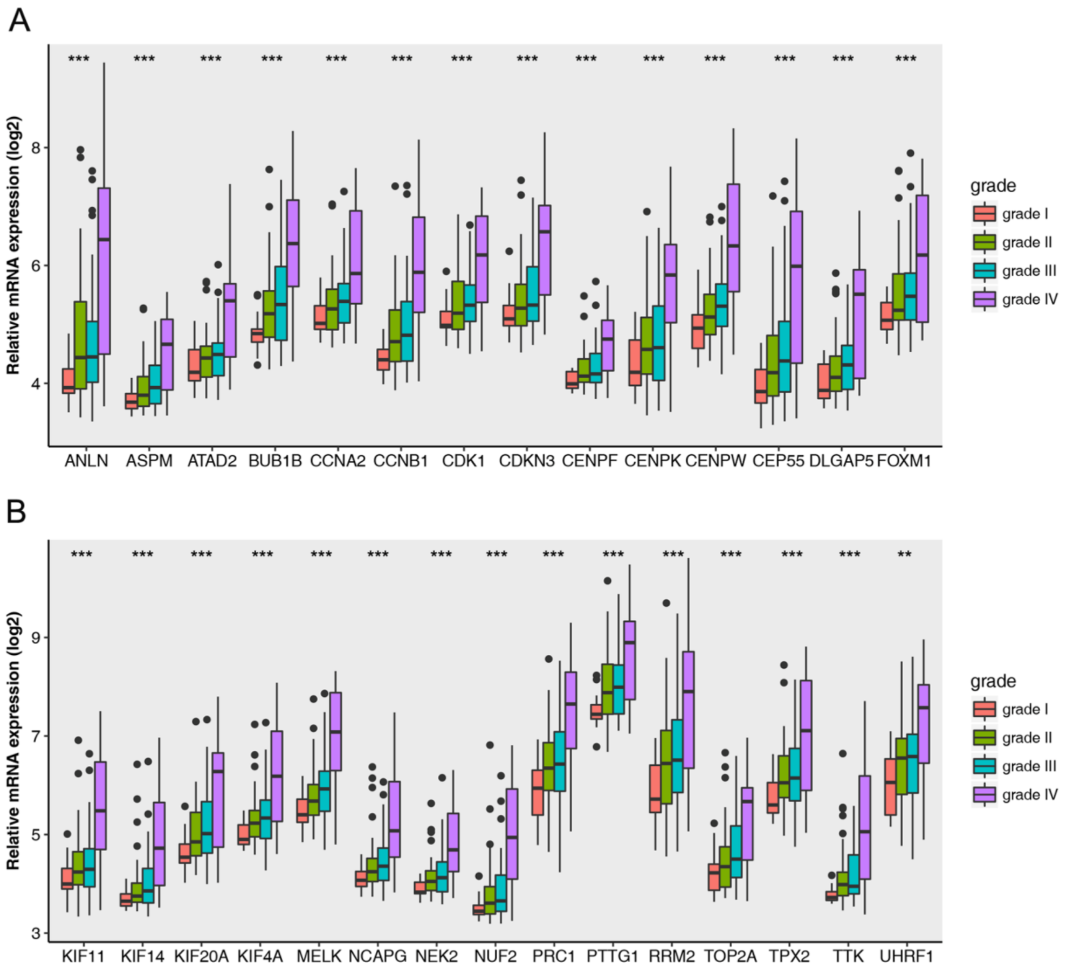


**Supplementary Figure S3.** Boxplots of hub genes across different tumor grades in the GSE73731. The boxplots show the medians and dispersions of the samples of different tumor grades for each hub gene. P values are the results of one-way ANOVA for different tumor grades. * $p < 0.05$, ** $p < 0.01$, *** $p < 0.001$.
