# Supplementary material for: Identification of Biomarkers Associated With Pathological Stage and Prognosis of Clear Cell Renal Cell Carcinoma by Co-expression Network Analysis
Source: Front Physiol. 2018 Apr 18;9:399. doi: 10.3389/fphys.2018.00399 (PMC5915556; doi:10.3389/fphys.2018.00399)
Supplement: Supplementary file 4 [file Image4.pdf]

Supplementary Figure S4

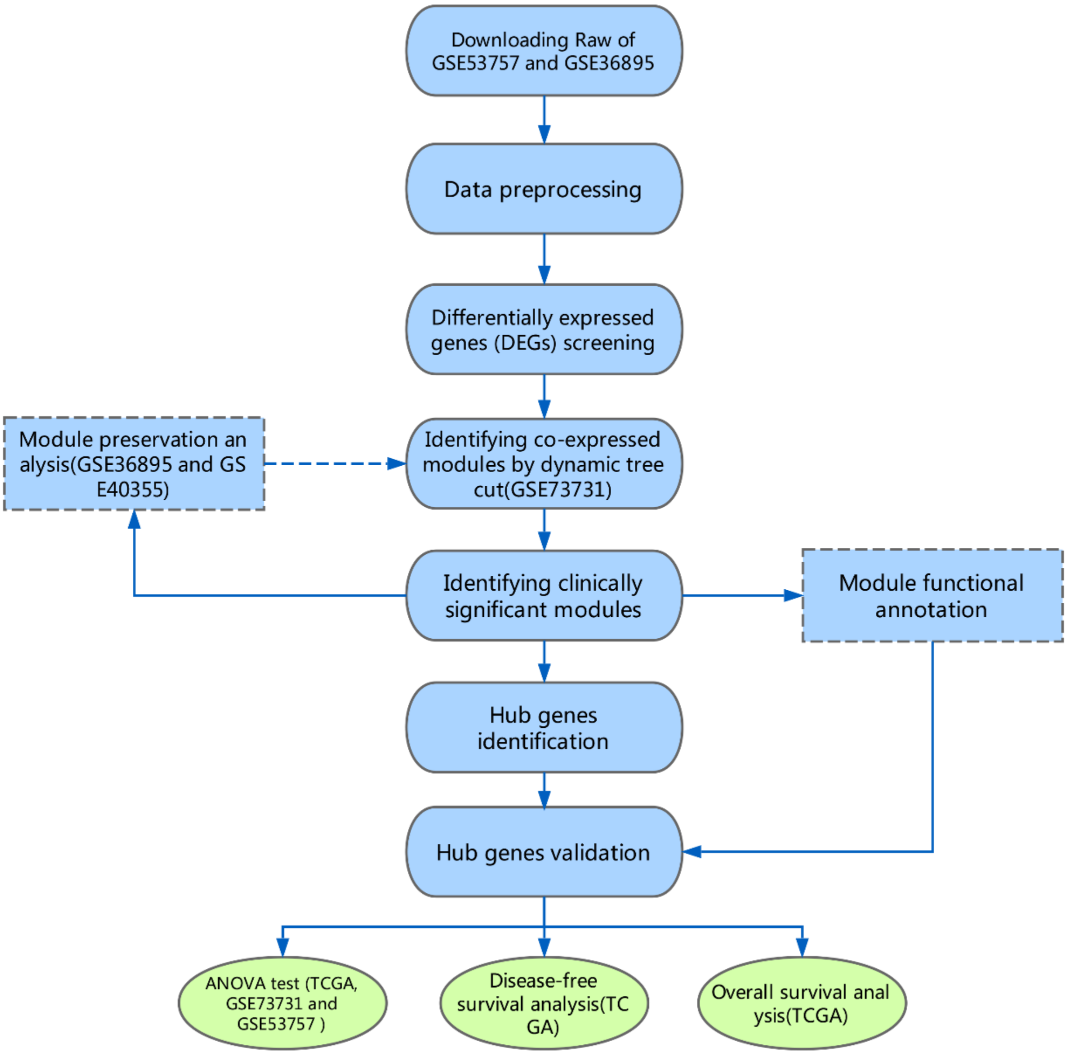

Supplementary Figure S4. Flow chart of data preparation, processing, nalysis and validation in this study.
