# Supplementary material for: Identification of Biomarkers Associated With Pathological Stage and Prognosis of Clear Cell Renal Cell Carcinoma by Co-expression Network Analysis
Source: Front Physiol. 2018 Apr 18;9:399. doi: 10.3389/fphys.2018.00399 (PMC5915556; doi:10.3389/fphys.2018.00399)
Supplement: Supplementary file 5 [file Table1.DOCX]

**Supplementary Table S1. Detailed information of datasets used in this study.**

| **GEO Accession** | **GSE53757** | **GSE36895** | **GSE73731** | **GSE40355** | **TCGA dataset** |
| --- | --- | --- | --- | --- | --- |
| **Platform** | Affymetrix HG U133 Plus 2.0 | Affymetrix HG U133 Plus 2.0 | Affymetrix HG U133 Plus 2.0 | Agilent-026652 | IlluminaHiseq |
| **Sample number** |  |  |  |  |  |
| Total | 144 | 76 | 265 | 48 | 530 |
| ccRCC | 72 | 29 | 265 | 16 | 530 |
| pStage I | 24 | - | 41 | - | 267 |
| pStage II | 19 | - | 12 | - | 57 |
| pStage III | 14 | - | 28 | - | 123 |
| pStage IV | 15 | - | 44 | - | 83 |
| Stage unknown | 0 | - | 140 | - | 0 |
| Normal kidney | 72 | 23 | 0 | 8 | 0 |
| Others | 0 | 0 | 0 | 24 | 0 |
