# Supplementary material for: Identification of Biomarkers Associated With Pathological Stage and Prognosis of Clear Cell Renal Cell Carcinoma by Co-expression Network Analysis
Source: Front Physiol. 2018 Apr 18;9:399. doi: 10.3389/fphys.2018.00399 (PMC5915556; doi:10.3389/fphys.2018.00399)
Supplement: Supplementary file 6 [file Table2.DOCX]

**Supplementary Table S2. Genes in the module are listed in gene-module.**

| Gene Module | Gene Symbol |
| --- | --- |
| Black module | ACOT6, ACOX2, ACSL5, ADAM28, ADAMDEC1, ADAP2, ADCK2, ADCY7, ADORA3, ADTRP, AIM2, AKIP1, AKNA, ALOX5, ALOX5AP, ANKRD44, ANXA1, AOAH, AP1S2, APBB1IP, APOBEC3C, APOBEC3G, APOC1, APOE, APOL6, AREG, ARHGAP25, ARHGAP30, ARHGAP9, ARHGEF6, ARL11, ARNT2, ARPC1B, ASS1, ATP2B4, AXL, BCAT1, BCL11B, BCL2A1, BCL2L10, BEX1, BEX2, BMP2K, BST2, BTN2A2, BTN3A1, BTN3A2, BTN3A3, C10orf128, C16orf54, C1orf162, C1QA, C1QB, C1QC, C3AR1, C5AR1, CA8, CACHD1, CARD16, CASP1, CCL18, CCL4, CCL5, CCR2, CCR5, CCRL2, CD14, CD163, CD2, CD247, CD27, CD300A, CD300LF, CD37, CD38, CD3D, CD4, CD40, CD48, CD52, CD53, CD69, CD72, CD74, CD84, CD86, CD8A, CD97, CECR1, CELF2, CFD, CHN2, CHST11, CLEC12A, CLEC2B, CLEC2D, CLEC4A, CLEC5A, CLEC7A, CMPK2, CMTM3, CMTM4, CNPY3, CORO1A, COTL1, CR1, CRLF3, CRTAM, CSF1R, CSF2RA, CSF2RB, CSRNP3, CST7, CTSS, CTSZ, CXCL10, CXCL11, CXCL13, CXCL16, CXCL5, CXCL9, CYAT1, CYBB, CYTH4, CYTIP, DAGLB, DAPP1, DCLK1, DDX58, DEF6, DEPTOR, DNASE2, DOCK10, DOCK2, DOCK8, DOK3, DOPEY2, DPEP2, DTX3L, DUSP4, EFHD2, EHBP1L1, ELF4, EMB, EMILIN2, EMP3, EMR2, EOMES, EPB41L3, EPSTI1, ERAP1, ERAP2, EVI2A, EVI2B, FAIM3, FAM129A, FAM171A1, FAM26F, FAM49A, FCER1G, FCGR1B, FCGR2A, FCGR2B, FCGR2C, FCGR3B, FCRL3, FERMT3, FGD2, FGR, FKBP11, FPR1, FPR3, FRK, FXYD5, FYB, GAPT, GBP1, GBP2, GBP4, GBP5, GCAT, GCH1, GLIPR1, GLIPR2, GLOD5, GLUL, GM2A, GNB4, GNLY, GPNMB, GPR171, GPR183, GPR34, GPR65, GPR82, GSTO2, GZMA, GZMB, GZMH, GZMK, H2AFY, HCK, HCLS1, HCP5, HIVEP3, HLA-B, HLA-C, HLA-DMA, HLA-DMB, HLA-DOA, HLA-DPA1, HLA-DPB1, HLA-DPB2, HLA-DQB1, HLA-DRA, HLA-E, HLA-F, HLA-G, HLA-J, HMOX1, HPSE, HS3ST2, HSPA6, IDO1, IER5, IFI27, IFI44, IFI44L, IFNAR2, IFNGR1, IGHG1, IGJ, IGK, IGLC1, IGLV1-44, IGSF6, IKZF1, IL10RA, IL10RB, IL12RB1, IL15RA, IL16, IL18BP, IL2RB, IL2RG, IL7R, ILDR2, INPP5D, IRAK3, IRF1, IRF7, IRF8, ISG15, ISG20, ITGAL, ITGAM, ITGAX, ITGB2, ITGB2-AS1, ITK, ITPR2, JAK3, JAZF1, KCNMB2, KIAA0226L, KIAA1551, KIF9, KLHL6, KLRB1, LAG3, LAIR1, LAMP3, LAPTM5, LAT2, LCK, LCP1, LCP2, LGALS9, LILRA2, LILRB2, LILRB4, LOC374443, LPAR5, LPXN, LRRC2, LRRK1, LSP1, LST1, LY86, LY96, LYN, LYZ, MAFB, MAP3K8, MBNL1, MCTP1, MDFIC, MFNG, MICB, MILR1, MIS18BP1, MNDA, MORN4, MPEG1, MR1, MS4A14, MS4A4A, MS4A6A, MSC, MSR1, MST1, MST1L, MYO1F, MYO1G, NAMPT, NCF1, NCF2, NCF4, NCKAP1L, NFATC2, NHSL1, NINJ2, NINL, NKG7, NLRC3, NLRC4, NLRC5, NMI, NPL, NRP2, OAS1, OAS2, OAS3, P2RX7, P2RY12, P2RY13, PARP14, PARP8, PARVG, PCED1B, PHGDH, PIK3R5, PKIB, PLA2G7, PLAC8, PLAUR, PLEK, PLEKHO1, PLXNC1, PNPLA4, PPM1K, PPP1R18, PRF1, PRKAR2B, PRKCB, PROC, PROX1, PRR15L, PSMB10, PSMB8, PSMB9, PSTPIP2, PTPN22, PTPRC, PVRIG, PYCARD, PYHIN1, RAB27A, RAB31, RAB33A, RAB37, RAC2, RAP1GAP, RAP2B, RAPGEF3, RASGEF1A, RASSF5, RCSD1, RENBP, RGS1, RGS10, RGS18, RGS19, RHOH, RMST, RNASE6, RNF166, RNF186, RRAD, RSAD2, RTP4, RUNX3, S100A8, S100A9, SAMD3, SAMD9L, SAMHD1, SAMSN1, SASH3, SCRN2, SDC3, SDS, SELENBP1, SELL, SELPLG, SEMA6D, SERPINB9, SFMBT2, SH2D1A, SIGLEC1, SIGLEC10, SIGLEC7, SIRPG, SKIDA1, SLA, SLAMF7, SLAMF8, SLC11A1, SLC15A3, SLC1A3, SLC7A7, SLC8A1, SLC9A9, SLCO2B1, SMAP2, SNX20, SP110, SP140L, SPATS2L, SRGN, STAB1, STAT1, STK10, STK17B, STX11, SYT11, SYTL3, TAGAP, TAP1, TAP2, TAPBP, TBC1D13, TBXAS1, TES, THEMIS, THEMIS2, TIFA, TLR1, TLR2, TLR7, TLR8, TM6SF1, TMC8, TMEM144, TNFAIP3, TNFRSF1B, TNFRSF9, TNFSF13B, TNFSF8, TRAC, TRAF3IP3, TRAT1, TRBC1, TRDV3, TREM2, TRIM22, TRPV2, TSPAN13, TSPAN4, TUB, TYMP, TYROBP, UBE2L6, UCP2, VAMP5, VAV1, VDR, VMP1, VNN2, VSIG4, WIPF1, XAF1, XCL1, ZNF267 |
| Brown module | ABCG1, ACOT8, ACSF2, ACTG2, ADAMTS5, ADCY3, ADM, ADSSL1, AFAP1L1, ALDOC, ANGPT2, ANGPTL2, ANGPTL4, ANO1, ANXA9, APCDD1, APEH, APLN, APOLD1, AQP1, ARHGEF10, ATP6V0E2, AVPR1A, BTC, C10orf10, C2orf40, C3orf70, C8orf4, CALCRL, CALD1, CCDC102B, CCDC85A, CCL28, CCND1, CD200, CD93, CDH13, CDH5, CDKN2B, CLEC14A, CLMN, COL15A1, COL21A1, COL4A1, COL4A2, COL5A3, CPA3, CPE, CPNE2, CR2, CRIP1, CSPG4, CXorf36, CYGB, DNAH11, DNER, DOCK9, DUSP1, EBF2, ECSCR, EDIL3, EDN1, EDNRA, EDNRB, EHD2, EHD3, ELK3, ELMO1, ELTD1, EMCN, EPHA3, ESM1, FAM101B, FILIP1, FJX1, FLT1, FMNL3, FOLH1B, FOXF1, FRZB, GABRD, GJA1, GJA4, GJC1, GNG11, GPR176, GPR4, GPT2, GREB1, GRIK3, GRK5, GUCY1A3, GUCY1B3, HAPLN1, HECW2, HES4, HEY1, HEY2, HEYL, HIGD1B, HILPDA, HLX, HMCN1, HOGA1, HOXD8, HSPG2, INHBB, ITGB1, JAG1, JAG2, JUN, KALRN, KCNAB1, KCNE3, KCNE4, KCNJ2, KCNN2, KDR, KIAA1462, LAMA4, LAMC1, LAYN, LDLRAD4, LHPP, LOC285812, LOC643733, LRP4, LRRC17, LURAP1L, LZTS1, MALL, MAP2, MAPRE2, MCAM, MEF2C, MFSD3, MMRN1, MMRN2, MPZL2, MUC13, MYADM, NETO2, NEURL1B, NFASC, NID1, NOTCH3, NOTCH4, NPNT, NPTX2, NPY1R, NR2F1, NR5A2, NRARP, NRG3, OLFML2A, OR51E1, OR51E2, PABPC4L, PALD1, PARM1, PBX1, PCCB, PCDH17, PCDHB14, PDGFD, PECAM1, PGF, PGM2L1, PHLDA1, PIEZO2, PIK3R3, PLAC9, PLAT, PLCL1, PLEKHB1, PLVAP, PLXDC1, PLXND1, PMEPA1, PNP, PODXL, PPP1R1A, PRKCA, PRSS23, PTP4A3, PTRF, QPRT, RAPGEF5, RASD1, RBP7, RERGL, RGCC, RGS3, RGS5, RHOJ, RNF180, S1PR1, SCD5, SCN4B, SDSL, SEMA3F, SEMA5A, SEMA5B, SIPA1L2, SLC14A1, SLC16A14, SLC1A4, SLC27A3, SLC39A14, SLC39A4, SLC6A1, SORT1, SOX11, SPARC, SPARCL1, SPHK2, SPRY1, ST6GALNAC2, ST6GALNAC3, STC1, STEAP4, STRIP2, SYNM, TCF4, TGFBR3, TLE1, TM4SF18, TMEM91, TPSAB1, TPSB2, TRAF3IP2, TRIM36, TSPAN33, UNC5B, VASH1, VEGFA, VWF, WASF3, WLS, YBX3, YWHAH, ZNF532 |
| Green module | AASS, ABCA1, ACOT13, ACOX1, ADAL, AGR2, ALDH1A2, ALS2CL, AMPH, ANKRD2, APOBEC3F, ARHGAP26, ARL4D, ARSF, ASAP1, ASB9, ATL3, BAZ1A, BMP7, C3orf58, C9orf135, CADM4, CALML3, CAND2, CANX, CASP4, CASR, CAV2, CCBE1, CDK19, CDO1, CHML, CHSY1, CLDN16, CLDN19, CLK4, CLUL1, COL4A6, COMMD8, CORO1C, CPNE6, CSGALNACT2, CYP3A4, DARS, DDN, DDX25, DDX26B, DEGS1, DERL1, DIAPH2, DISC1, DLEU2, DLGAP2, DPP6, ECHDC1, EDDM3A, EGFR, EGLN1, ELF5, ENAM, EOGT, ERO1L, ESR1, ESRRB, FAM13A-AS1, FANCL, FAS, FEM1C, FIGNL1, FMN2, FNIP1, GABRP, GAS5, GIT2, GLS, GNA13, GNB5, GP2, GRHL2, GSAP, HACL1, HCG4, HECW1, HPCAL4, HRASLS2, HS2ST1, IFI16, IGH, IGSF11, IKBIP, INSIG2, JMY, KCNK10, KCTD3, KCTD9, KDELC2, KDM3A, KIAA1715, KIF16B, KLK6, KLK7, KSR2, LGALS8, LINC00887, LOC100130872, LOC149703, LOC153682, LOC728040, LPGAT1, LRRC43, LXN, LYPD6B, MAP3K9, MAP4K4, MAP6, MAPK1, MCUR1, MFAP3, MLKL, MOGAT1, MRAP2, MTHFD2L, MYCN, MYLK3, MYOF, NACA, NAP1L1, NAT2, NBN, NCK1, NKAIN4, NLK, NOS1, NPHS1, NR1I3, NR3C1, NTNG1, NXPH2, OGFRL1, OVOL2, PAG1, PGK1, PIK3C2G, PLCD4, PLD1, PLIN5, PM20D1, PMP22, PPM1E, PPP1R3B, PPP2R2B, PROM2, PROZ, PSMA5, PTPN13, RAB38, RAB8B, RASL11B, RDH12, RECQL, RELL1, RGS8, RIMBP2, RND3, RNF149, RNF217, RNF219, RP2, RPA1, S100A14, SACS, SCUBE3, SEMA3B, SERPINA4, SERPINB1, SERTAD2, SFR1, SGPP1, SIM2, SLC13A3, SLC22A13, SLC25A33, SLC28A2, SLC2A12, SLC30A2, SLC30A8, SLC30A9, SLC38A1, SLC47A2, SLC4A9, SLC52A3, SLC5A11, SLFN11, SLFN12, SLFN13, SLFN5, SMYD2, SNORD89, SORCS1, STAMBPL1, STRA6, SUCLG2, SYN2, SYNE4, SYPL2, TAF9B, TCAIM, TCL6, THRSP, TMC4, TMEM87B, TMPRSS4, TNFAIP8, TNNT2, TPK1, TPTEP1, TRNT1, TRPC1, TRPM6, TYRO3, USP46, VIM, WIF1, YME1L1, ZAK, ZEB2, ZFAS1, ZNF292, ZNF582-AS1 |
| Greenyellow module | ACOT12, ACPP, ADH1C, AFM, ALB, AQP2, CALB1, CDKN1C, CLIC5, CLSTN2, CRHBP, CRYAA, CYP27B1, CYP2B6, DIO1, DPEP1, ENPP6, FABP1, FGF1, FLJ22763, FLJ35700, FXYD4, GPR110, GSTM3, HPD, HS6ST2, IRX1, IRX2, IYD, KRT7, LINC00551, LOC284578, NPHS2, OLFM4, PAPPA, PLA2R1, PLCXD3, PTPRO, RALYL, REEP6, S100A2, SFRP1, SFXN2, SLC12A1, SLC13A2, SLC16A5, SLC22A8, SLC26A4, SLC34A1, SLC5A2, SLC7A13, SOST, TAC1, TACSTD2, TCEAL2, TCF21, TMEM178A, TMEM207, TNNC1, UMOD, UPP2, XPNPEP2 |
| Magenta module | ACSS2, AMBP, ANXA2R, APOC3, APOH, ARHGDIB, ARRDC2, ATP2C2, C6orf123, CARD8, CAV1, CCND2, CD1D, CD36, CLIC2, CSMD1, CXCL12, CXCR4, CYP17A1, CYP4F2, CYP4F3, CYSLTR1, DOCK4, ENTPD1, ETS1, FAM124A, FAM198B, FAM65B, FAR2, FLI1, GGTA1P, GIMAP1, GIMAP2, GIMAP4, GIMAP6, GIMAP7, GMFG, GNG2, GPR155, HHEX, ITGA4, KLHDC9, LIPA, LZTS3, MARCKS, MS4A7, NAB1, OAT, P2RY8, PAQR7, PCSK1N, PELI1, PLEKHA2, PLXNB1, PNPLA3, PPP1R16B, PRDM1, PREX1, PRKCH, PRTG, PTPRE, RASSF2, RASSF3, RFTN1, RNF125, RNF157-AS1, SETD7, SGK2, SH2B3, SLC51B, ST8SIA4, SWAP70, TLR4, TMED4, TOX2, TSC22D3, VEGFC, WFDC2 |
| Pink module | ABCA8, ACTN1, ADAM12, ADAMTS2, ADH1B, C1orf216, C1R, C1S, C3, C7, CAMK1D, CCDC64, CD99, CDH11, CDKN1A, CEBPB, CHST15, CHSY3, CLDN11, COL1A1, COL1A2, COL3A1, COL5A1, COL5A2, COL6A2, COL6A3, COL8A1, COLGALT1, CTGF, CTHRC1, DCN, FAP, FBLN1, FBLN5, FBN1, FLNA, FN1, FSCN1, FSTL1, GAS1, HK1, HRH1, HSPA2, IGFBP2, IL4R, INHBA, ITGA5, ITPRIPL2, KCNS3, LGALS1, LINC00924, LOXL2, LUM, MAN1C1, MAP1B, MAP3K7CL, MARVELD1, MEST, MOXD1, MRGPRF, MT1E, MT1F, MT1G, MT1H, MT1HL1, MT1M, MT1X, MT2A, MYH10, NAP1L3, OGDHL, OLFML2B, OSMR, PAX8, PCDH7, PDGFRA, PHLDA2, PLP2, POSTN, PRR16, PXDN, SCG2, SERPINE1, SERPINH1, SHC1, SLC2A3, SLC43A3, SLIT2, SMIM3, SOCS3, SPINK13, SULT1C2, TAGLN2, TGFB1, TGFBI, TGM2, TIMP1, TIMP2, TMEM173, TMEM2, TUBA1A, TUBB6, VCAN, VEPH1 |
| Purple module | ALDH1B1, ATP1A1, C1orf116, CA10, CCDC160, CCDC181, CHL1, DCXR, DHRS11, DIRAS3, DUSP9, EMX1, F11, FAM167A, GABRA2, GATA3, GGT6, GPC5, HRG, KCNE1, KCNJ1, KCNJ10, KIAA2022, KLHL14, KNG1, LHX1, LINC00645, LINGO2, LOC100130691, LOC400043, LPPR1, MAGI2-AS3, MAGI2-IT1, MRO, MUC15, NRK, OTOGL, PAPPA2, PCDH9, PDE1A, PGR, PPAPDC1A, PTH1R, RANBP3L, RASSF10, SALL3, SCN2A, SCN7A, SERPINA5, SHC3, SLC12A3, SLC15A2, SLC7A8, SOSTDC1, SOWAHA, SUCNR1, SUSD4, TBC1D24, TFAP2B, TMEM45B, TMEM52B, TNFSF15, TUBB2B, TYRP1, WNK4, ZNF503 |
| Red module | ADORA2B, AIF1L, ANKRD36, ANKRD36B, ANLN, ANXA2, APAF1, APOBEC3B, APOL1, ARL4A, ARL4C, ASPH, ASPM, ATAD2, B3GNT5, BAG2, BCAM, BCL6, BEND7, BICD1, BID, BIRC3, BIRC5, BRIP1, BSPRY, BTNL9, BUB1, BUB1B, C15orf48, C18orf54, C3orf67, C6orf223, CALU, CARS, CASC5, CCDC109B, CCDC88A, CCL20, CCNA2, CCNB1, CCNB2, CCNE2, CCSAP, CD44, CDC7, CDCA7, CDK1, CDKN3, CENPA, CENPE, CENPF, CENPH, CENPK, CENPW, CEP55, CKLF, CNDP1, COLEC11, CSTA, CTNNAL1, CTTN, CYFIP2, DDX60L, DLGAP5, DNAJC12, DNASE1L3, DPY19L1, DTL, DUSP5P1, E2F7, E2F8, ECT2, EFHD1, EIF4EBP1, EPB41L1, EPM2A, EZH2, FABP5, FANCI, FGD6, FGFBP2, FGFR2, FMO4, FNDC3B, FOLR1, FOXM1, FRAS1, GGH, GINS2, GPX8, H2BFXP, HAUS6, HGF, HOMER1, HPS3, IBSP, ICAM1, IDH1, IGF2BP3, IGFBP1, IL13RA2, IL1RL1, IL20RB, IL4I1, IL7, ITPR3, KDELC1, KIAA0101, KIAA0930, KIF11, KIF14, KIF20A, KIF23, KIF2A, KIF4A, KLF15, KNTC1, KRBA1, KSR1, LHFPL2, LY75, MAD2L1, MASP1, MCM5, MDH1B, MELK, METTL7A, MMP1, MMP9, MPZL1, MTHFD2, MYBL1, NAPSA, NCAPG, NCAPG2, NDC80, NEK2, NFE2L3, NOD2, NPC1, NSMAF, NT5E, NUCB2, NUF2, NUSAP1, OSBPL6, PALM, PBK, PELI2, PLOD2, PRC1, PRIM1, PRR11, PSAT1, PTGER3, PTTG1, RAB42, RACGAP1, RAD51AP1, RASA3, RCN1, RFC2, RFX2, RHBDF2, RNF145, RNF213, RPL22L1, RRM2, SCG5, SEC24D, SEMA3G, SGOL2, SHMT1, SLC16A1, SLC16A10, SLC16A6, SLC17A4, SLC26A6, SLC2A1, SLC41A2, SLC44A4, SLC6A18, SLC7A11, SMC4, SPAG5, SPOCK1, STEAP3, SYBU, TCF19, TCIRG1, TFPI2, TMEFF2, TMEM132E, TMEM206, TMEM44, TMEM45A, TNFRSF11A, TOP2A, TPX2, TRIB3, TRIM59, TRIP13, TSPAN7, TTC39C, TTK, TUBB2A, UBE2C, UHRF1, WNK1, WT1, ZNF385B |
| Turquoise module | A1CF, ABAT, ABCB1, ACAA1, ACAD8, ACADM, ACADSB, ACAT1, ACE2, ACMSD, ACOT4, ACSM2A, ACSM2B, ACSM3, ACSS3, ACY3, ADA, ADH6, ADI1, AGBL3, AGMAT, AGPAT3, AGPAT9, AGXT2, AHCY, AIFM1, AK3, AKR1A1, AKR7A3, ALAD, ALDH1L1, ALDH2, ALDH3A2, ALDH4A1, ALDH5A1, ALDH6A1, ALDH7A1, ALDH8A1, ALDOA, ALDOB, AMDHD1, AMN, ANGPTL3, ANK2, ANK3, ANO4, ANPEP, AOX1, AP1M2, AP5M1, APOB, APOM, ARHGAP24, ASNS, ATP11A, ATP5G3, ATP6V1A, ATPIF1, ATXN7L1, AUH, AZGP1, BAG1, BASP1, BBOX1, BCKDHB, BDH2, BDKRB2, BDNF, BHMT, BHMT2, BPHL, C11orf54, C11orf71, C1orf115, C1orf210, C21orf33, C9orf66, CA2, CA4, CADPS2, CAPN3, CAT, CCDC110, CDCA2, CDH16, CDH6, CDHR2, CDK18, CDKL1, CDON, CDS1, CEACAM1, CENPV, CGN, CHDH, CHRDL1, CHST9, CLCN5, CLDN10, CLDN2, CLU, CLYBL, COBL, COBLL1, COL23A1, COL4A3, COL4A4, CORO2B, COX7B, CPEB3, CPEB4, CREB5, CRYL1, CRYM, CTD-3080P12.3, CTSH, CTXN3, CUBN, CXCL14, CYP2J2, CYP4A11, CYP8B1, CYS1, CYSTM1, DACH1, DAK, DAO, DBT, DDC, DEPDC7, DGCR5, DGKD, DHDH, DHRS4-AS1, DHTKD1, DIRAS2, DMGDH, DNAJC6, DOC2A, DPY19L2P2, DPYS, ECHDC3, ECHS1, ECI2, EHHADH, EMX2, EMX2OS, ENPP3, ENPP5, ENTPD5, EPB41L4A-AS1, EPB41L5, EPCAM, EPHX2, ERMP1, ESPN, ESRRG, ETNK2, ETNPPL, EVA1C, FABP3, FAM110C, FAM151A, FAM184A, FARP1, FBP1, FBXL16, FBXO21, FCAMR, FCGR1A, FDX1, FECH, FGFR3, FHL1, FKBP10, FLRT3, FMO1, FREM2, FTCD, FUT3, FUT6, FXYD2, FZD1, G0S2, G6PC, GABBR1, GABRB3, GAL3ST1, GALM, GATM, GBA3, GCHFR, GDA, GGACT, GHR, GIPC2, GJB1, GLTPD2, GLYAT, GLYCTK, GOT1, GPD1, GPD1L, GPHN, GPX3, GRAMD1C, GRHPR, GRIA4, GRTP1, GSTA1, GULP1, GXYLT2, HADH, HAGH, HAO2, HAVCR1, HDHD3, HGD, HHLA2, HIBADH, HIBCH, HINT2, HMGCL, HMGCS2, HNF4A, HRSP12, HSD11B2, HYAL1, IDNK, IGF2BP2, IL17RB, IL17RD, IQGAP2, KCNH6, KCNJ15, KCNJ16, KCNK5, KCNMA1, KCTD6, KHK, KIAA1161, KITLG, KL, KLHDC7A, KLHL13, L1CAM, L2HGDH, LDHB, LDLR, LEAP2, LGALS2, LIME1, LIN52, LINC00173, LINC00462, LINC00472, LINC00537, LINC00622, LINC00671, LINC00955, LOC100422737, LOC389332, LRP2, LRRC16A, LRRC19, LRRK2, LYPLAL1, MACROD1, MAGI1, MAGI2, MAOA, MAP7, MARVELD2, MARVELD3, MCCC2, MDH1, MECOM, MFSD4, MGAM, MICALL2, MIOX, MME, MORN2, MPC1, MPP5, MRPL34, MSRA, MTCH2, MTFP1, MUC20, MUT, MYH8, MYO3A, MYO5B, MYO6, MYO7B, N4BP2L1, NAPEPLD, NAT8B, NAV1, NAV2, NCOA7, NDUFS1, NEBL, NEDD4L, NEGR1, NKIRAS1, NLGN1, NNT, NOX4, NPR3, NQO2, NR3C2, NTN4, NUDT6, OCLN, OGG1, OR7E14P, PAIP2B, PALM3, PANK1, PAQR5, PBLD, PCCA, PCK1, PCK2, PCYT2, PDHA1, PDHB, PDP2, PDZD3, PDZK1, PDZK1IP1, PDZRN3, PEBP1, PEG3, PEPD, PER2, PGPEP1, PHB, PHYH, PHYHIPL, PIP5K1B, PIPOX, PKHD1, PKLR, PLA2G12B, PLCB1, PLEKHA6, PLG, PLLP, PNMA2, PNPO, PP7080, PPARA, PPARGC1A, PRAP1, PRODH2, PRSS8, PRUNE2, PTGFRN, PTGR1, PTPN3, PXMP2, QDPR, QRFPR, RAB11FIP3, RAB17, RAB27B, RASSF6, RBM47, RBP4, RBP5, RDH11, REN, RGN, RGS7, RHOBTB3, RIMKLA, RIT1, RNF152, RNF165, RUNX2, SAMD5, SAT2, SC5D, SCGN, SEMA6A, SEPP1, SERPINA6, SETD3, SH3YL1, SHISA9, SLC10A2, SLC12A6, SLC13A1, SLC16A9, SLC17A1, SLC1A1, SLC22A11, SLC22A12, SLC22A2, SLC22A6, SLC22A7, SLC23A1, SLC23A3, SLC25A10, SLC25A48, SLC25A5, SLC26A9, SLC27A2, SLC2A2, SLC2A4RG, SLC2A9, SLC38A4, SLC39A5, SLC4A4, SLC51A, SLC5A10, SLC5A12, SLC6A13, SLC6A19, SLC6A3, SLC7A9, SMLR1, SMTNL2, SORD, SOX6, SPATA17, SPATA18, SPIRE1, STRADB, SUCLG1, SUOX, SUSD2, SVIP, SYNE1, TEX11, THRB, THSD7A, TINAG, TLN2, TLR3, TM7SF2, TMED6, TMEM106A, TMEM139, TMEM140, TMEM174, TMEM252, TMEM27, TMEM72, TNFRSF11B, TNFSF9, TOX3, TPMT, TREH, TRHDE, TRHDE-AS1, TRIM2, TRIM55, TRIM6, TRNP1, TRPM3, TSPAN1, TST, TTC38, UCHL1, UGT2B28, UGT3A1, UNC5CL, UPB1, UQCRFS1, USP2, VAT1L, VAV3, VIL1, VWA8, WDFY3-AS2, WDR72, ZBED5-AS1, ZMYND12, ZNF204P, ZNF320, ZNF395, ZNF667-AS1, ZNRF3, ZYG11A |
| Yellow module | ABCC1, ABCC3, ABHD14B, ABHD17C, ACLY, ACOT11, ADRB1, AGXT, AHCYL2, AHR, ANGPTL1, ANO5, ANXA4, APOO, AQP6, ATP6V0A4, ATP6V0D2, ATP6V1B1, ATP6V1C2, ATP6V1G3, BDH1, BHLHE41, BMPR1B, BNIP3, BSND, C16orf89, C19orf33, C1orf168, C1orf226, C2orf88, C3orf52, C4orf47, C6orf136, C9orf24, C9orf84, CA9, CASZ1, CDCA7L, CDH3, CEBPD, CEL, CKMT2, CLCNKB, CLDN8, CLNK, CNTN3, COPG2IT1, CP, CRNDE, CWH43, DANCR, DDB2, DDIT4, DEFB1, DMRT2, DNASE1, EGF, EGLN3, EHF, ENO1, ENO2, EPB41L4B, EPN3, ERBB4, ERP27, ERV3-2, ERVMER34-1, ESRP1, FA2H, FAM169A, FAM216A, FAM222A, FAM3B, FAM57A, FAM83B, FGF9, FOLR3, FOXI1, FREM1, FUT11, GABARAPL1, GAS2L3, GATA3-AS1, GCDH, GLRX5, GMPR, GOT2, GPC3, GPR98, GRIK2, GRM1, HEPACAM2, HIGD1A, HK2, HMX2, HOXB-AS3, HPCAL1, HSD3B7, HSPB8, HYKK, ID2, IDH2, IGFBP3, IL32, ILDR1, INPP5J, IRF6, IRX3, KCTD1, KLHL3, KLK1, LARS2, LDHA, LDHD, LDLRAD3, LMO3, LOC154761, LOX, LPCAT1, MAGI3, MAL, MCOLN3, MDM2, MIR210HG, MPP7, MPPED2, MRPS25, MYC, MYO3B, NDUFA4, NDUFA4L2, NEK6, NNMT, NOL3, NR0B2, OVOL1, OXCT1, P4HA1, P4HB, PACRG, PAK6, PCP4, PDK1, PDLIM1, PFKFB2, PFKFB3, PFKP, PFN2, PHKA2, PHYHD1, PIGR, PLAGL1, PLCG2, PLEKHA5, PLK2, PPFIA4, PPM1H, PRDM16, PRDX4, PRKCDBP, PRLR, PROCR, PRR15, PRRG2, PTGER1, PVALB, PYGL, RAB11FIP4, RAB25, RALGPS1, RHBG, RHCG, RNASET2, RNF150, RPS6KA6, S100A10, SAMD12, SAP30, SCARB1, SCNN1A, SEC61G, SEL1L3, SERPINA1, SETMAR, SH3GL2, SHISA2, SHMT2, SIM1, SIRPA, SIX4, SLC15A4, SLC16A7, SLC25A25, SLC25A5-AS1, SLC26A7, SLC35G2, SLC4A1, SLC4A11, SMIM5, SMPD1, SNRK-AS1, SOD3, SPAG4, SPTBN2, SRD5A3, SSPN, ST3GAL6, STAP1, STC2, STK33, STMN3, SUSD3, TBC1D1, TBX3, TFAP2A, TFCP2L1, TGFA, TMCC1, TMEM116, TMEM213, TMEM30B, TMEM61, TMPRSS2, TMSB10, TNFAIP6, TPD52L1, TPM1, TRIM50, TRIM9, TTC36, TYMS, UGT1A1, UNC5D, UQCRC1, VTCN1, WDR54, WNK3, YEATS2, ZC3HAV1L |
| Grey module | ABCA17P, ABCB4, ABI3BP, ACO1, ACSL6, AFF3, AHNAK2, AMFR, AMT, ANKRD37, ANXA3, APOD, AQP11, ARG2, ARHGEF26, ARL10, ATHL1, BCHE, BCL11A, BIK, BRE-AS1, BTD, C10orf99, C12orf56, C14orf37, C16orf58, C4orf6, C5, C5orf46, C8orf22, CCR6, CD70, CDH4, CDH9, CDKN2A, CHGB, CHI3L1, CHODL, CMC4, CNTN1, COCH, COL4A5, CPN2, CSDC2, CTH, CX3CR1, CXCL2, CYP24A1, CYP26B1, CYP39A1, DAB2, DCDC2, DNMT3L, DSP, EAF2, EFCAB4B, EFHC2, EML6, ENOX1, ENPP1, ERGIC1, EYA2, EYA4, FABP6, FABP7, FAM65C, FAM84A, FBXO16, FDXR, FER1L4, FERMT1, FGF11, FGG, FHIT, FHOD3, FMO5, FOXC1, FOXJ3, FSTL3, GABRB2, GAD1, GALNT3, GAS2, GAS6-AS1, GC, GCM1, GDPD3, GJA3, GK, GK3P, GLDC, GLRB, GLYATL1, GPM6B, GPR27, GPR37, GPR64, GRAMD1B, GRAMD4, GRB14, GRHL1, GRIN2A, H2BFS, HIST1H2BK, HLA-DQA1, HMGN5, HOXB6, HOXB8, HPGD, IP6K3, ISM1, KCNJ13, KCNK3, KISS1R, LAD1, LGSN, LINC00240, LINC00473, LOC389906, LONRF2, LPA, LPPR5, LRRC41, LY6E, MAL2, MAP9, MCHR1, MCTP2, MET, MTCP1, MTTP, MUC1, NAGS, NAP1L2, NCOR2, NDNF, NEFL, NELL1, NMB, NOG, NRN1, NTRK2, NUPR1, ODF3B, PAGR1, PC, PCBP2, PCDHB16, PCDHB6, PCSK6, PDE4A, PERP, PF4V1, PFKFB4, PGBD5, PHLDA3, PKM, PLA1A, PLCB4, PLEKHG4, PNCK, PNMAL1, POU5F1P3, POU5F1P4, PPP1R13L, PPP1R3C, PREX2, PRIMA1, PROM1, PRSS16, PSORS1C3, PTGDS, PTGS1, PTHLH, PTPRD, RALGAPA2, RARRES2, RASSF8-AS1, RDH10, RDH13, REEP1, REG1A, SCD, SCIN, SCN3A, SCNN1B, SCNN1G, SDC1, SEZ6L2, SHISA3, SIGIRR, SLC16A2, SLC19A2, SLC3A2, SLITRK5, SMCO3, SNAP25, SNCA, SOBP, SORCS3, SPINK1, STK39, STS, SYTL2, TENM2, TEX15, TIMD4, TMEM100, TNFRSF10B, TRIM63, TRPA1, TSPAN8, TUBAL3, UPK1B, VLDLR, VNN1, VSIG1, VSNL1, WWC3, ZDHHC2, ZGPAT, ZNF300P1 |
